# A Large Congenital Ventricular Outpouching

**Published:** 2019-01

**Authors:** Tahereh Davarpasand, Mohammad Sahebjam, Mohammad Alidoosti

**Affiliations:** *Tehran Heart Center, Tehran University of Medical Sciences, Tehran, Iran.*

**Keywords:** *Heart defect, congenital*, *Echocardiography*, *Echocardiography, three-dimensional*, *Heart ventricles*

A 64-year-old diabetic man with the complaint of an atypical chest pain of many years’ duration and a mildly positive myocardial perfusion scan was referred for coronary artery angiography. The patient’s electrocardiography showed a J-point elevation in the precordial leads. Transthoracic echocardiography (two- and three-dimensional) revealed localized thinning and an outpouching, which extended from the mid inferoseptal and anteroseptal wall of the left ventricle (LV) to the mid inferior wall with mild LV systolic dysfunction (Figure 1, Figure 2, Video 1, & Video 2).

**Figure 1:**
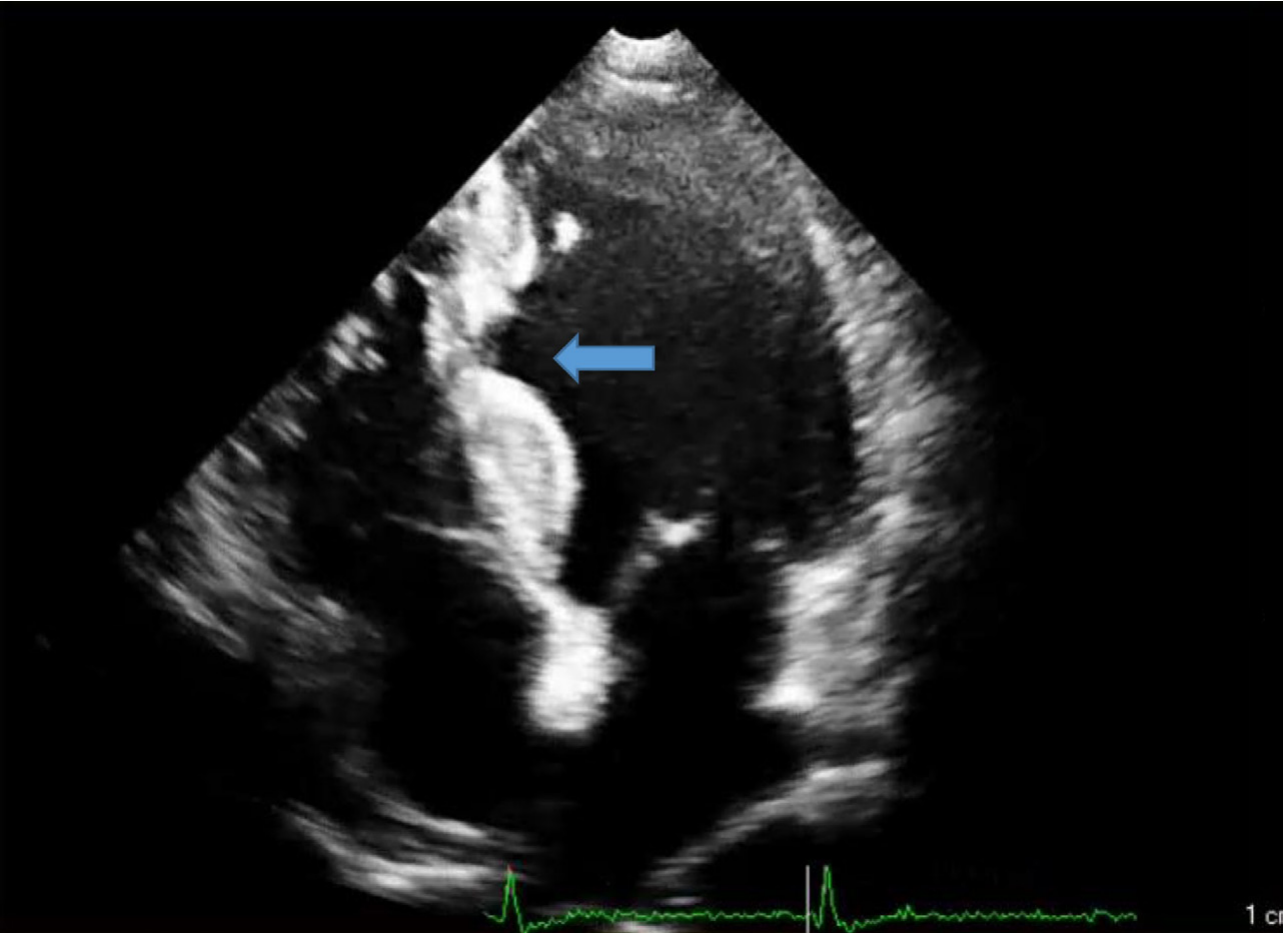
Four-chamber view of two-dimensional transthoracic echocardiography, showing a diverticulum in the mid inferoseptal wall (arrow)

**Figure 2:**
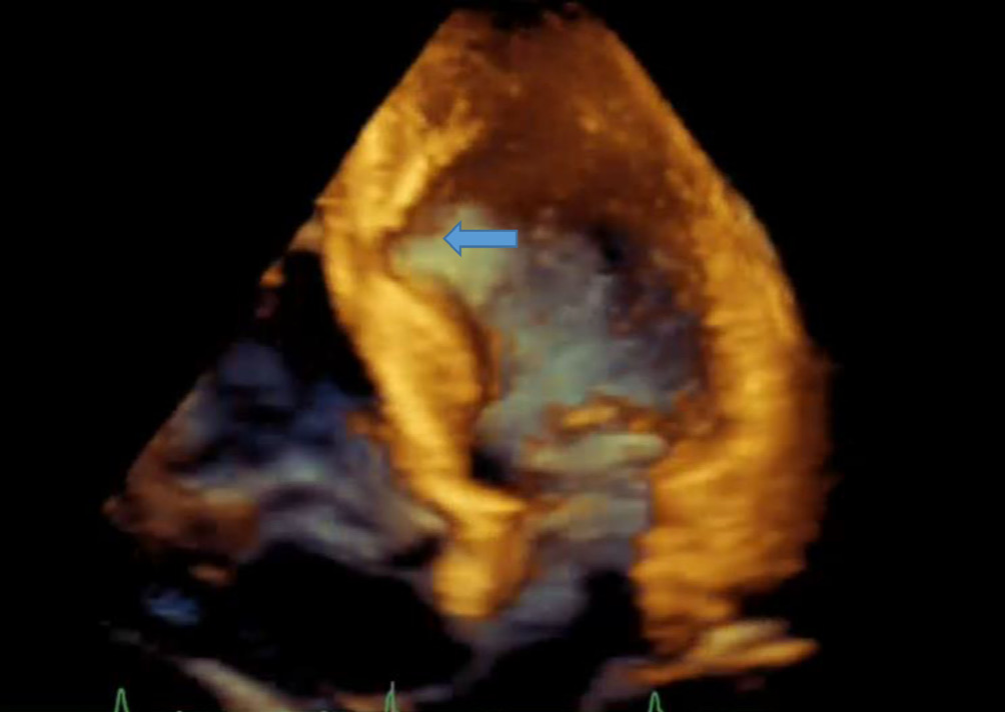
Four-chamber view of three-dimensional transthoracic echocardiography, showing a diverticulum in the mid inferoseptal wall (arrow)

 Coronary artery angiography showed normal coronary arteries, and ventriculography revealed a narrow-necked outpouching in the posterobasal LV wall with systolic contractions, suggestive of an asymptomatic huge ventricular diverticulum (Figure3). The patient refused to undergo cardiac magnetic resonance imaging despite our recommendation.

**Figure 3:**
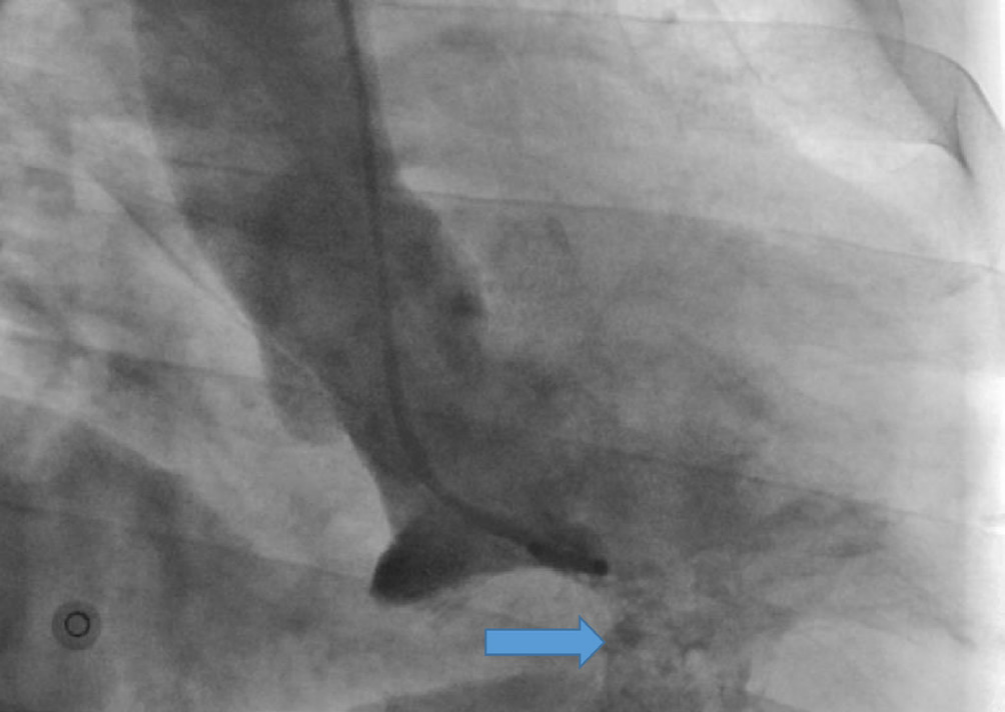
Right anterior oblique view of left ventricular angiography, showing a diverticulum in the posterobasal wall (arrow)

Congenital LV diverticula constitute a rare congenital disorder, especially in adulthood.^1^Although most of these diverticula are congenital in etiology, one can never be sure. Diverticula are more prevalent in Asia than are primary LV aneurysms and are associated with other cardiac and extra-cardiac anomalies in more than 30%of cases. The most common differential diagnosis is a ventricular aneurysm, which can be differentiated according to the morphology of the defect and the associated coronary artery disease in secondary type aneurysms.^1^^, ^^2^ Arrhythmias, emboli and strokes, ruptures, and even sudden cardiac deaths are the rare complications of diverticula. The management is done individually according to the patient’s symptoms and associated complications; however, there is not a documented outcome and a valuable long-term follow-up.^2^

***To watch the following videos, please refer to the relevant URLs:***

http://jthc.tums.ac.ir/index.php/jthc/article/view/934/821

Video 1. Left ventricular diverticulum in 2chamber view of transthoracic echocardiography

http://jthc.tums.ac.ir/index.php/jthc/article/view/934/822

Video 2. Enface view of left ventricular diverticulum in 3D transthoracic echocardiography.

## Notes:

***This paper should be cited as: ***Davarpasand T, Sahebjam M, Alidoosti M. A Large Congenital Ventricular Outpouching. J The Univ Heart Ctr 2019;14(1):42-43.
